# Isolation and characterization of a novel K3-type capsule-targeting phage for the treatment of carbapenem-resistant *Acinetobacter baumannii*

**DOI:** 10.1128/spectrum.01098-25

**Published:** 2025-11-05

**Authors:** Hewen Deng, Ziqiang Liu, Siyun Wang, Shitong Lu, Ruopeng Cai, Linwan Feng, Kun Shi, Xin Tan, Rui Du

**Affiliations:** 1 College of Animal Science and Technology, Jilin Agricultural University 85112, Changchun, China; 2 State Key Laboratory of Quantitative Synthetic Biology, Shenzhen Institute of Synthetic Biology, Shenzhen Institutes of Advanced Technology, Chinese Academy of Sciences 568741, Shenzhen, China; Peking University People's Hospital, Beijing, China

**Keywords:** *Acinetobacter baumannii*, bacteriophage, phage therapy, phage receptor

## Abstract

*Acinetobacter baumannii* (*A. baumannii*) is a prevalent pathogen associated with nosocomial infections, particularly carbapenem-resistant *A. baumannii* (CRAB), which poses a significant global public health concern. The rising prevalence of CRAB underscores the urgent need for novel therapeutic strategies, including phage-based interventions. Here, we isolated and characterized novel phage P11B exhibiting a potent lytic activity specifically against K3 capsule-type CRAB with an optimal multiplicity of infection of 0.0001, an exceptionally short latent period (~10 min), and a high burst size (~188 PFU/cell). P11B further demonstrated superior stability across wide temperature/pH ranges, enhancing therapeutic potential and surpassing many previously reported K3-specific phages. Phage-host co-cultivation experiments generated resistant mutants showing markedly attenuated virulence compared to wild-type strains. Whole-genome sequencing identified three key mutations in *pgi*, *algC*, and *galU* genes encoding metabolic enzymes and extracellular proteoglycan biosynthesis regulators. Genetic complementation restored phage susceptibility, confirming that these mutations mediate phage adsorption through capsular modification. Our study identifies the core genes that link phage receptor functionality with virulence in CRAB, demonstrating an evolutionary trade-off between phage resistance and pathogenicity. These findings elucidate the molecular mechanisms underlying phage-host interactions and the core genetic determinants that connect phage receptor modulation to virulence attenuation in CRAB, revealing an evolutionary trade-off that supports the use of capsule-targeted phage therapy against K3-type *A. baumannii* infections.

**IMPORTANCE:**

The global spread of MDR-AB, particularly carbapenem-resistant *Acinetobacter baumannii* (CRAB) strains with various capsule types, necessitates the development of innovative antimicrobial strategies. In this study, we identify phage P11B as a potent lytic agent against KL3 CRAB and reveal an evolutionary trade-off: mutations in capsule biosynthesis genes (*pgi*, *algC*, *galU*) that confer phage resistance also directly reduce virulence. This biological constraint addresses a significant limitation of phage therapy by ensuring that resistant mutants remain less pathogenic. Our findings mechanistically link modifications in phage receptors to a reduction in virulence, thereby advancing capsule-targeted phage engineering as a sustainable approach to combat CRAB. This study clarifies fundamental genetic factors that connect the modulation of phage receptors with the reduction of virulence in CRAB, highlighting an evolutionary compromise that enhances the therapeutic promise of phages aimed at the capsule in treating KL3-type *A. baumannii* infections.

## INTRODUCTION

*Acinetobacter baumannii* is currently recognized as a critical-priority MDR nosocomial pathogen and is included among the six highly pathogenic and antibiotic-resistant bacterial pathogens known as ESKAPE (1) (*Enterococcus faecium*, *Staphylococcus aureus*, *Klebsiella pneumoniae*, *A. baumannii*, *Pseudomonas aeruginosa*, and *Enterobacter* spp.). In recent years, the issue of drug resistance in *A. baumannii* has become increasingly severe (2, 3). This bacterium exhibits high resistance to multiple antibiotics, particularly broad-spectrum and multi-antibiotic agents, including carbapenem antibiotics (4). This resistance poses a significant challenge for clinical treatment, especially in intensive care units and trauma care units (5).

With the rise of carbapenem-resistant *Acinetobacter baumannii* (CRAB), these strains are becoming increasingly resistant to traditional antibiotics, complicating treatment and failing to meet clinical needs (6), and the development of new antibiotics is progressing at a significantly slower rate than the evolution of resistant bacteria (7, 8). Consequently, there is an urgent necessity to develop new or alternative therapies to effectively tackle the growing issue of antibiotic resistance. Phages are viruses that specifically target bacteria (9), and these phages typically infect only particular species or strains of bacteria, which positions them as a potentially effective therapeutic tool for combating bacterial infections. Their lower propensity to develop drug resistance makes them promising therapeutic agents for targeted bacterial infections (10). An increasing number of animal experiments have verified the safety and reliability of phage therapy (11–13), and more and more clinical attempts have also yielded positive results (14–16).

*A. baumannii* possesses various virulence factors, including outer membrane protein A (OmpA) (17), phospholipase D (18), capsular polysaccharides (19), and biofilm-associated protein (Bap) (20). These factors also act as phage adsorption receptors on the bacterial surface, playing a crucial role in the initial recognition and attachment of phages to the host (21), which determines the specificity and efficiency of phage-host interactions (22). Among these factors, capsular polysaccharides are particularly significant, as they enable bacteria to effectively evade or counteract the host’s immune response, and capsular polysaccharides have been demonstrated to serve as receptors for various bacteriophages (23). Furthermore, based on the diversity of capsular polysaccharides, *A. baumannii* strains can be classified into multiple capsule types (24). *A. baumannii* can produce structurally distinct capsule polysaccharides (i.e., K types), with biosynthesis determined by the gene content of capsule synthesis loci (KL types). Therefore, KL provides a genetic subdivision that complements the phenotypic K type classification. Up to now, at least 237 K-locus (KL) reference sequences (representing >65 K-types) have been identified. Dominant capsule types include K3, K2, K9, K13, and K49 (25, 26). Among these, notably, K3 capsule type strains exhibit higher clinical prevalence, augmented pathogenicity, and elevated multidrug resistance (26). Consequently, we selected the K3 capsule type strain as the research host to isolate phages and further investigate the relationship between phage receptor mutations, phage sensitivity, and bacterial virulence. Recent studies have shown that mutations in phage receptors on the bacterial surface have a significant impact on the sensitivity of bacteria to phages (22, 27), significantly affecting the efficacy of phage therapy in clinical settings. Conversely, receptor mutations can restore strain sensitivity to antibiotics and reduce bacterial virulence (28). These findings emphasize the dual role of phage receptors as both barriers and portals in phage therapy, providing new insights for the clinical treatment of MDR bacterial infections. Understanding how to specifically manipulate the trade-off between bacterial resistance to phages and bacterial virulence, as well as identifying key genes, will greatly assist in clinical treatment.

In this study, we collected clinical isolates of *A. baumannii* and utilized the specialized K3 strain TAB11B as host to isolate lytic phage from sewage samples. We characterized the growth characteristics and observed the morphology of the phages. The lytic spectrum test demonstrated that the phages exhibited a strong lytic activity capable of lysing all specific capsular types of *A. baumannii*. These characteristics suggest the potential of this phage as an alternative to antibiotics for the clinical treatment of *A. baumannii* infections. Through the co-culture of phages and their host, we selected phage-resistant strains and identified the key genes for the phage receptors via sequencing comparisons. This research provides both a material foundation and theoretical insights for the clinical application of phages.

## MATERIALS AND METHODS

### Bacterial strains and phage isolation

The *A. baumannii* strain TAB11B was generously provided by Sun Yat-sen University and currently maintained in our laboratory. A total of 143 clinical CRAB isolates were kindly provided by Dr. Yongjun Pan from Southern University of Science and Technology Hospital, Dr. Changqing Bai from Shenzhen University General Hospital, Dr. Hongzhou Lu from Shenzhen Third People’s Hospital, Dr. Jinzhu Mao from The First Affiliated Hospital of Guangzhou Medical University, Dr. Wei Huang from Shenzhen People’s Hospital, and Prof. Yinyue Deng from Sun Yat-Sen University. ATCC 17978 was obtained from our laboratory collection. Detailed information for each isolate is documented in Table S1. All bacterial strains were cultured in Luria-Bertani (LB) broth (Lennox, HKM, Guangdong, China), LB semisolids containing 0.7% agar or on LB agar at 37°C. Phage P11B was isolated from sewage water samples collected from medical sewage systems in several hospitals in Shenzhen, China, utilizing TAB11B as the host strain. Briefly, 2 mL of sewage filtered through 0.22 µm was mixed with 1 mL of 3× LB broth, and 1 mL of host bacteria in the logarithmic growth phase (OD = 0.6) was added. The culture was cultured at 37°C and 220 rpm for 6–8 h. The culture was centrifuged at 5,000 × *g* and filtered through 0.22 µm. Spot tests were used to detect the presence of phage (29), and phage was purified via repeated double-layer agar plate method (30) until the plaques on the plate were uniform in size and morphology. The counting of phage was performed using the double-layer agar plate method (30).

### Host range determination

The host range of P11B was tested against 144 strains using spot tests (29). Briefly, 100 µL of overnight bacterial cultures was mixed with molten soft agar (0.7% [wt/vol]) and overlaid onto solidified LB agar plates. After solidification, 10 µL of P11B suspensions was spotted onto the agar surface and allowed to absorb completely. The plates were then incubated aerobically at 37°C overnight, followed by an analysis of plaque formation.

### Growth characteristics of P11B

After cultivating TAB11B to the late logarithmic growth phase, it was diluted with fresh LB broth (2 × 10^7^ CFU/mL), and phage P11B was added at a multiplicity of infection (MOI) of 0.01. This co-culture was then transferred to a 96-well plate and incubated at 37°C with shaking for 16 h. Absorbance was measured at 600 nm using a microplate reader, and the data collected over the aforementioned time period were used to plot the growth curve. Additionally, a control was set up without the addition of phage. The assay was repeated three times.

The MOI refers to the ratio of phages to host bacteria during the infection process. After culturing TAB11B to the logarithmic growth phase, it was transferred to fresh LB liquid medium at a final concentration of 10^7^ CFU/mL. Subsequently, phage P11B was added at various MOIs (phage/bacteria = 0.00000001, 0.0000001, 0.000001, 0.00001, 0.0001, 0.001, 0.01, 0.1, 1, 10, 100) and incubated in a constant-temperature shaking incubator for 6 hours at 37°C and 220 rpm. The phage titers of the samples were then determined immediately using the double-layer agar plate method following serial dilution (30). The assay was repeated three times.

For the determination of the one-step growth curve, P11B was introduced to an exponential phase TAB11B culture (1 × 10^7^ CFU/mL) at an MOI of 0.1 and allowed to adsorb for 10 min at 37°C. Subsequently, the mixture was centrifuged at 12,000 × *g* for 5 min at 4°C, and the resulting pellet was resuspended in 10 mL of LB broth. The suspension was then incubated at 37°C with shaking at 220 rpm. The co-cultures were collected at 10 min intervals until 150 min had elapsed. Finally, the titers of the co-cultures were quantified using the double-layer agar plate method. The assay was repeated three times.

### Stability studies of P11B

To assess the stability characteristics of P11B, the survival rate was evaluated following treatment across a range of pH levels and temperatures. Specifically, for the pH stability tests, aliquots of phage suspensions were incubated at 37°C across a pH spectrum of 1 to 14 for 1 h. In the thermal stability tests, aliquots of phage suspensions were incubated at 4, 20, 37, 42, 50, 60, 70, and 80°C for 1 h, with samples collected at 30 min intervals until the total incubation reached 60 min. All treated samples were subsequently diluted and immediately tested using the double-layer agar plate method. The assay was repeated three times.

### Concentration, purification, and electron microscopy observation of P11B

High concentrations of P11B were obtained using PEG precipitation methods (31) and subsequently purified by chlorinated cesium (CsCl) density gradient ultracentrifugation (CsCl gradients: 1.10, 1.30, 1.50, and 1.70 g/mL). The phage concentrate was then dialyzed against SM buffer to remove cesium chloride from the phage sample, resulting in a highly concentrated and pure phage suitable for transmission electron microscopy (TEM). The morphology of P11B was examined using a TEM (HITACHI HT-7700, Japan) at an acceleration voltage of 80 kV.

### Sequencing and analysis of the phage genome

The phage genome of P11B was extracted from the concentrated phage, as purified above, using a viral DNA kit (Omega Bio-Tek, Inc., Doraville, GA, USA). Whole-genome sequencing was subsequently performed by Novogene Co., Ltd. (Beijing, China), utilizing the Illumina NovaSeq X Plus sequencing platform. The phage genome was assembled using MEGAHIT software (v1.2.9), followed by open reading frame (ORF) prediction, functional annotation, and gene classification using Pharokka software (v1.7.2). The genome of phage P11B was visualized using the online tool PHASTEST (https://phastest.ca/) (32). The sequences encoding the terminase large subunit of phage P11B were compared using BLAST to identify homologous phages. The nucleic acid sequences of these selected phages were then obtained and compared using the ClustalW program. Subsequently, these sequences were utilized to construct a neighbor-joining phylogenetic tree with 1,000 bootstrap replicates, employing MEGA11 software (33).

### Isolation of phage-resistant bacterial mutants

Phage P11B and the host strain TAB11B were co-incubated overnight until complete lysis, indicated by the clarity of the solution, followed by bacterial regrowth, evidenced by the restoration of turbidity. Single colonies were isolated by streaking on LB agar plates. After being cultured alternately in the LB liquid medium and LB agar medium twice, a phage-tolerant bacterial variant was obtained. The efficiency of plating (EOP) assay was performed to confirm the susceptibility of the isolated strains to P11B. All phage-resistant strains were stored frozen at −80°C in 30% glycerol.

### Characterization of phage-resistant strains

After sequencing and analyzing the phage-resistant strains (BioProject: PRJNA1296866), a comparison was made with the genome of the wild-type *A. baumannii* TAB11B to identify gene mutations that may confer phage resistance. After confirming that the mutant gene was related to capsular polysaccharide by sequencing, the capsule and lipopolysaccharide of the strain were extracted according to the method of Pan (34). After separation by 12% sodium dodecyl sulfate-polyacrylamide gel electrophoresis (SDS-PAGE), they were visualized by Alcian blue staining (34). After an overnight culture, the *pgi*-mutant, *algC*-mutant*,* and *galU*-mutant were diluted to a ratio of 1:400, and the optical density at 600 nm (OD600) was measured to construct a growth curve.

### *Galleria mellonella* virulence assays

To compare the virulence of phage-resistant mutants with that of the parental strain TAB11B, we utilized *Galleria mellonella* larvae as an infection model. This model has been employed to assess microbial pathogenicity and virulence across various bacterial pathogens (35, 36). *Galleria mellonella* larvae measuring 2.0 to 2.5 cm in length were injected with a bacterial suspension of *A. baumannii* TAB11B, as well as mutant strains *pgi*-mutant, *algC*-mutant, and *galU*-mutant (10^8^ CFU/mL, 10 µL per larva) into the left hindmost proleg using a micro sample syringe (25 µL) from Sangon Biotech Co., Ltd. (Shanghai, China). The control group larvae were injected with an equal volume of phosphate-buffered saline (PBS) only, and the blank group was not treated with any treatment, with 20 larvae in each group. The larvae were then incubated at 37°C in the dark, and the number of survivors was monitored every 12 h for up to 72 h. The virulence of each strain was assessed by analyzing the survival curves.

### Scanning electron microscopy

To observe the morphological changes of the mutant strains compared to the wild-type strain, strains were cultivated to the exponential phase (OD600 = 0.4–0.6) and subsequently washed three times with sterile PBS (13). After being immobilized in 4% glutaraldehyde overnight, the strains were dehydrated using ethanol at varying concentrations (20, 50, 70, 90, and 100%) and then freeze-dried on cover glasses. Finally, the bacterial surface morphology was examined using scanning electron microscopy (SEM) (Hitachi SU8010, Hitachi Scientific Instruments, Japan).

### Complementation assay to confirm target gene mutation-dependent phage resistance phenotype

To confirm that the observed phage resistance phenotype was specifically caused by the target gene mutation, plasmid-based complementation experiments were performed. The wild-type allele of the target genes (*pgi*, *algC*, *galU*) was amplified by PCR using primers listed in the supplemental material and subsequently cloned into the pAb-Apr plasmid vector under the control of the inducible promoter TetR utilizing a seamless cloning kit (TransGen Biotech Co., Ltd., catalog number: CU201-02, Beijing, China). The recombinant plasmid (pAb-Apr-*pgi*, pAb-Apr-*algC*, pAb-Apr-*galU*) and the empty vector pAb-Apr were separately introduced into the *pgi*-mutant, *algC*-mutant, and *galU*-mutant strains via electroporation transformation. The transformed cells were selected on LB agar supplemented with 50 µg/mL apramycin sulfate and verified by colony PCR and Sanger sequencing. The EOP assay was performed to confirm the susceptibility of the complemented strains (*pgi*/pAb-Apr-*pgi*, *algC*/pAb-Apr-*algC*, *galU*/pAb-Apr-*galU*), vector control (*pgi*/pAb-Apr, *algC*/pAb-Apr, *galU*/pAb-Apr), wild-type TAB11B, and *pgi*-mutant, *algC*-mutant, and *galU*-mutant strains to P11B.

### Statistical analysis

The data are expressed as mean ± standard deviation. All experiments were conducted independently in triplicate. Statistical analyses were performed using GraphPad Software (GraphPad Software Inc., San Diego, CA, USA).

## RESULTS

### Phage isolation

**Fig 1:**
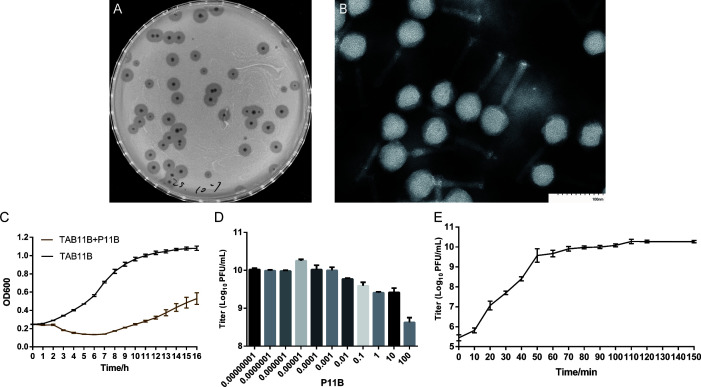
Biological characteristics of P11B. (**A**) Plaque of the purified bacteriophage; (**B**) observation of P11B by TEM; (**C**) growth curves of TAB11B with P11B treatment; (**D**) titers of P11B under different MOIs; and (**E**) one-step growth curve of P11B.

Clinical strain *A. baumannii* TAB11B was utilized as a host to isolate phages from sewage samples. The results obtained from the spot tests indicated a successful isolation of phages. After conducting five to six rounds of the double-layer agar plate method for purification, phages exhibiting clear plaques with uniform sizes and distinct boundaries were obtained and named P11B (Fig. 1A). The purified phage was introduced into the TAB11B bacterial culture. After incubation until the lysate became clear, it was filtered using a 0.22 µm microporous filter membrane. Subsequently, the titer of P11B was determined using the double agar method, yielding a concentration of 2 × 10^9^ PFU/mL.

### Host range of P11B

Among the 144 *A*. *baumannii* strains screened, phage P11B formed clear spots on the lawns of all 13 strains of the K3 capsule type, including TAB11B (see Table S1). This finding indicates that these strains are sensitive to this phage. Conversely, other capsular types of *A. baumannii* strains stored in the laboratory do not exhibit sensitivity to this phage (see Table S1). The plaque formed by P11B on lawns was surrounded by an enlarged halo, indicating that this phage likely encodes a depolymerase with polysaccharide-degrading activity (37–39).

### Morphological observation of P11B

TEM results showed that the virus particles of phage P11B feature an icosahedral head with a diameter of approximately 60 nm, alongside a tail measuring about 130 nm in length and 10 nm in width (Fig. 1B). These observations suggest that P11B morphologically belongs to the *Myoviridae* family.

### Characterization of phage P11B

The growth curve indicates that phage P11B effectively inhibits the growth of TAB11B for approximately 8 h (Fig. 1C). However, after this period, the OD600 value of the co-culture solution begins to rise, suggesting the emergence and rapid proliferation of bacteriophage-resistant mutant strains. When the MOI was set at 0.00001, the titer of the proliferated phage P11B reached its peak, approximately 1.8 × 10^10^ PFU/mL (Fig. 1D). The one-step growth curve experiment indicated that the latent period for P11B was approximately 10 min (Fig. 1E), after which the number of released viral particles increased rapidly, peaking at around 40 min with a high burst size of approximately 188 PFU/cell, suggesting a rapid and efficient host lysis.

### Stability studies of P11B

**Fig 2:**
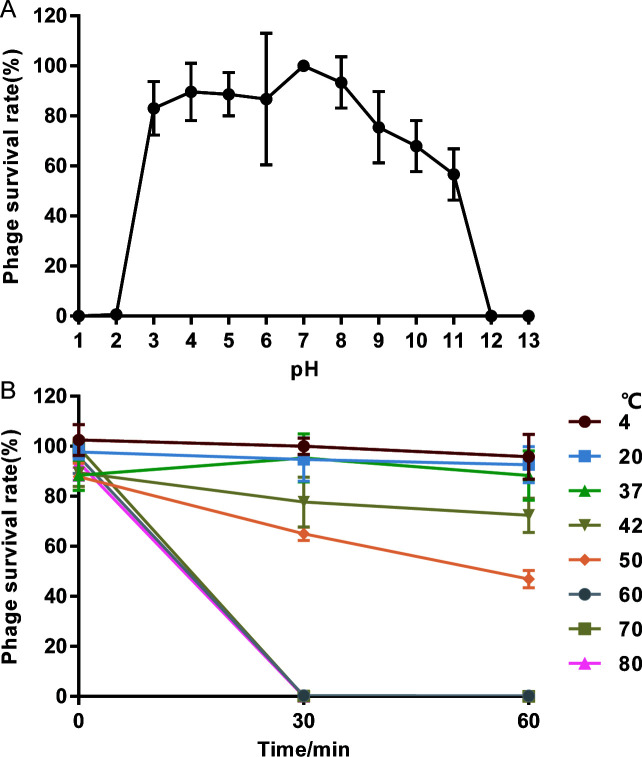
Stability tests of P11B. (**A**) pH stability. (**B**) Thermal stability.

The pH and thermal stability profiles of phage P11B were assessed under varying conditions. The titer of P11B remains relatively stable within the pH range of 3 to 9, with a phage survival rate exceeding 80%. This indicates a broad acid-base tolerance. However, the titer decreases sharply under extreme acidic (pH 1–3) or alkaline (pH 11–14) conditions (Fig. 2A). The titer of P11B can be maintained when the temperature is between 4 and 37°C. However, at a temperature of 42°C, the survival rate of P11B drops to less than 80%. Furthermore, when the temperature reaches or exceeds 50°C, the activity of the phage is significantly reduced (Fig. 2B).

### Overview of phage P11B genome

**Fig 3:**
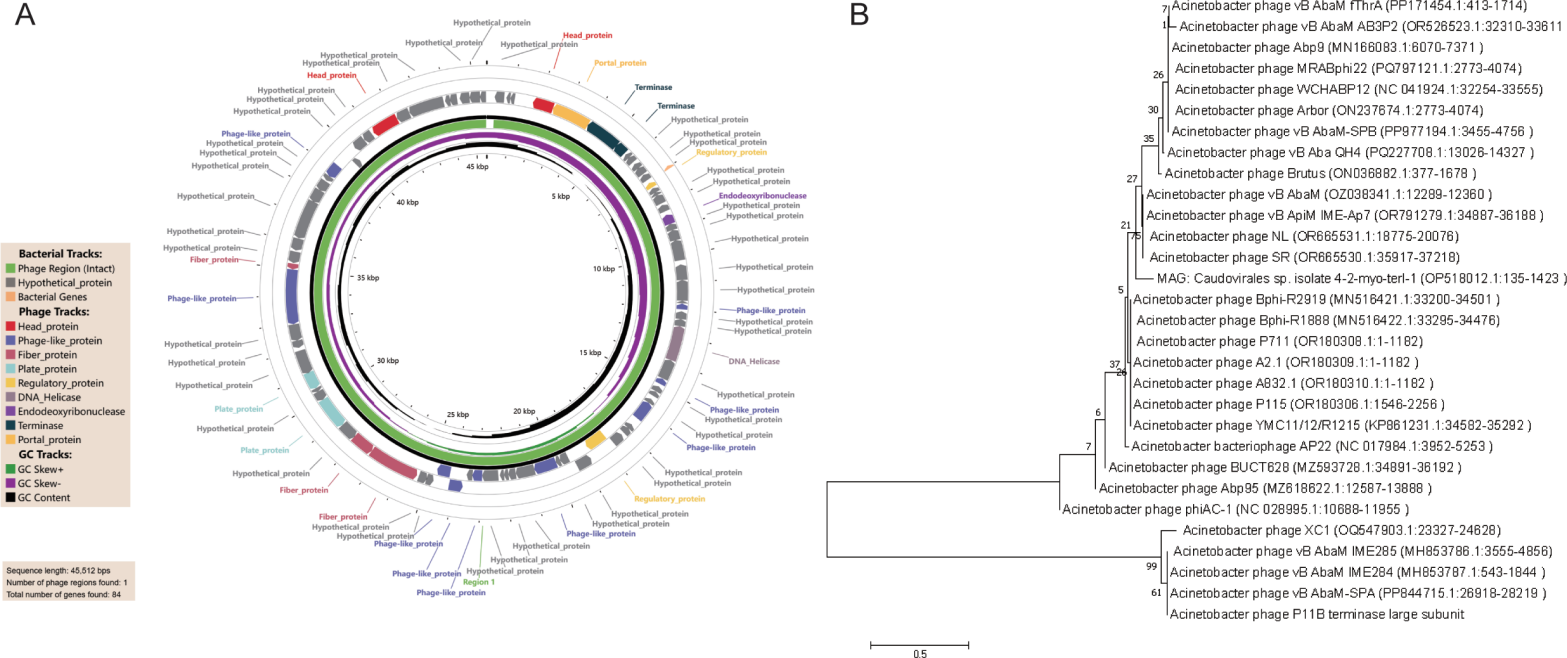
Bioinformatics analysis. (**A**) Genetic and physical organization of the P11B; linear genome of P11B depicted in a circularized format. A total of 83 ORFs are shown by the direction of transcription. (**B**) Neighbor-joining phylogenetic tree based on the terminase large subunit genes of P11B and related sequences. Bootstrap values > 50% (based on 1,000 replicates) are shown at the branch points. GenBank accession numbers are given in parentheses following phage.

The genome sequence of phage P11B has been submitted to the GenBank database under accession number PV851422. Sequencing results indicated that P11B possesses a double-stranded genome of 45,512 bp with a guanine and cytosine content of 37.87% (Fig. 3A). Based on the results from BLASTn, with coverage exceeding 70% and identity surpassing 90% (40), the analysis of the full-length genome of P11B against the GenBank database revealed a significant homology with the genomes of *Acinetobacter* phages Abp9, MRABphi22, AB_SZL3, vB_AbaM_IME512, P919, and AbP2 (online resource). Notably, nucleotide BLAST results showed that the genome does not have 100% identical sequence homologies to any currently recognized phage strains in GenBank, suggesting that it represents a novel phage strain. Furthermore, the genome analysis of P11B identified 83 genes, with the longest ORF measuring 2,031 bp. The phylogenetic analysis of the terminase large subunit gene revealed that P11B shares a close evolutionary relationship with several *Acinetobacter* phages, including vB AbaM-SPA, vB AbaM IME284, vB AbaM IME285, and XC1 (Fig. 3B).

### *pgi***,** *algC***,** and *galU* gene mutations confer phage resistance in bacterial strains

**Fig 4:**
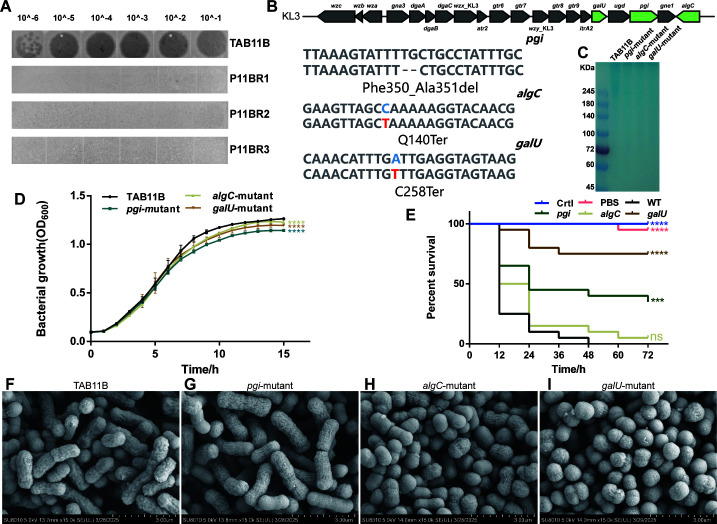
Characterization of the phage-resistant strains. (**A**) EOP assay of the wild-type strains and phage-resistant bacterial mutants on P11B. (**B**) Mutation details of the target gene of the mutant strain. (**C**) After being separated by 12% SDS-PAGE, the CPS phenotypes of TAB11B, *pgi*-mutant, *algC*-mutant, and *galU*-mutant were visualized by Alcian blue staining. (**D**) Growth curves of mutants and wild-type strains. (**E**) Virulence test: survival curve of the *Galleria mellonella* infection experiment*. pgi: pgi-*mutant*; algC: algC-*mutant*; galU: galU-*mutant. (**F–I**) SEM analyses of the surface morphology of TAB11B, *pgi*-mutant, *algC*-mutant, and *galU*-mutant (***P < 0.001; ****P < 0.0001; “ns” means not significant).

Phage-resistant strains were identified through co-cultivation. The EOP assay demonstrated that the mutants had lost sensitivity to phage P11B (see Fig. 4A). Sequencing analysis of three strains revealed that, compared to the wild-type strains, base mutations occurred in the *pgi*, *algC*, and *galU* genes. The specific types of mutations and their details can be seen in Fig. 4B. These three mutants were designated as *pgi*-mutant, *algC*-mutant, and *galU*-mutant, respectively. The *pgi, algC*, and *galU* genes have been reported to be associated with capsule synthesis in *A. baumannii* (41–44), indicating that the strains *pgi*-mutant, *algC*-mutant, and *galU*-mutant may have lost their capsules. Therefore, we employed the Alcian blue staining method to determine the capsule status of these strains. The results indicated that the staining bands of the *pgi*-mutant, *algC*-mutant, and *galU*-mutant strains were very light in color. In contrast, the wild-type strain TAB11B exhibited a high molecular weight Alcian blue-stained substance at the top of the SDS-PAGE gel (see Fig. 4C). Thus, it can be concluded that the capsule synthesis in the mutant strains is significantly affected. The growth curves of the strains *pgi*-mutant, *algC*-mutant, and *galU*-mutant were compared to that of the wild-type strain TAB11B. The analysis revealed differences between the mutant strains and the wild-type strain, suggesting that the mutations in the relevant genes have an impact on bacterial growth (see Fig. 4D). The virulence of the mutant strains was evaluated in comparison to TAB11B using the *Galleria mellonella* infection model. Mutant strains exhibited reduced virulence (see Fig. 4E), particularly the *galU* mutant, which resulted in only 20% mortality within 24 hours and maintained a 75% survival rate by the conclusion of the 72 hour test period. However, the analysis of survival rate of the *algC*-mutant strain compared to the WT strain showed no significant difference (*P* value: 0.0889), indicating that the impact of different genes on bacterial virulence varies. Further observation through SEM revealed that the surfaces of the three mutant strains (see Fig. 4G to I) were rough and wrinkled, in contrast to the smooth surface of the strain TAB11B (see Fig. 4F). Notably, unlike the rod-shaped morphology of TAB11B and *pgi*-mutant, the majority of the *algC*-mutant and *galU*-mutant strains exhibited a morphology similar to cocci, indicating that the deletion of *algC* and *galU* can affect the morphology of the bacteria. These results suggest that phage-resistant mutants exhibit pleiotropic effects, such as diminished fitness and reduced bacterial virulence, which may provide clinical advantages in addressing pathogenic CRAB infections.

### Restoration of P11B susceptibility by complementation in phage-resistant mutants

**Fig 5:**
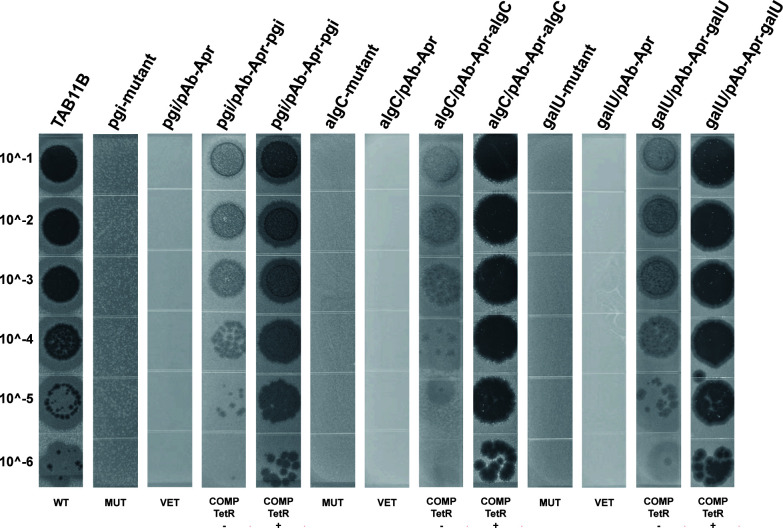
EOP assay of the wild-type strain (WT), phage-resistant strains (MUT), vector control strains (VET), complemented strains induced by TetR (COMP TetR+), and complemented strain not induced by TetR (COMP Tet R−) on P11B.

The target genes *pgi*, *algC*, and *galU* were amplified using PCR, subsequently cloned into a vector, and electroporated to generate the complement strains. The successful construction of these strains was confirmed through Sanger sequencing. The results of the EOP experiments conducted on wild-type strain TAB11B (WT in Fig. 5), mutant strains (MUT in Fig. 5), empty vector control strains (VET in Fig. 5), and complemented strains (COMP in Fig. 5) demonstrated that the complementation of the target gene restored sensitivity to phage P11B (see Fig. 5). In comparison to mutant and empty vector plasmid strains, the complemented strain exhibited a significant enhancement in EOP. Interestingly, the EOP of the phage on the complemented strain not induced by TetR (COMP TetR− in Fig. 5) was also restored, but both the EOP and the transparency of the formed plaques were lower than those of the induced complemented strains (COMP TetR+ in Fig. 5). This may be attributed to the slight expression of the target gene resulting from the incomplete shutdown of the induction system. Another noteworthy observation is that the plaques of the strain with the *pgi* complement as the host are not as distinct as those of the other two strains. This observation may be attributed to the role of *pgi* in encoding phosphoglucose isomerase, which plays a critical role in sugar metabolism. Following complementation, it is possible that this alteration impacts the host’s energy supply, subsequently affecting the efficiency of phage replication and resulting in less transparent plaques. According to the above observations, the complementing genes (*pgi*, *algC*, and *galU*) play a crucial role in restoring phage receptors on the bacterial surface, thereby facilitating phage attachment and subsequent infection.

## DISCUSSION

The emergence of CRAB has necessitated the exploration of alternative therapeutic strategies (45), with phage therapy emerging as a promising approach (46). Previous treatment cases have underscored its potential (15). In this study, we isolated and characterized a potent lytic phage, P11B, which demonstrated significant lytic activity against clinically isolated CRAB strains. Through co-culture experiments, analysis of phage-host interactions, and investigation of receptor recognition, we provided mechanistic insights into the therapeutic potential and limitations of this phage.

We utilized all clinical isolates stored in the laboratory as test bacteria to assess the lysis spectrum of phage P11B. Among these isolates, 13 strains of capsule type K3 exhibited sensitivity to the phage and demonstrated a robust lytic activity, highlighting the specificity of the phage. However, the extremely high specificity of phages presents limitations for clinical applications and phage therapy, as demonstrated by P11B, which exhibits no lytic activity against other capsular types of clinical *A. baumannii*. On the other hand, the one-step growth curve analysis of the phage revealed a short period of 10 min and a high burst size of approximately 188 PFU/cell, indicating a rapid and efficient host lysis. The optimal multiplicity of infection (MOI) is 0.0001, which indicates that a minimal number of phages can effectively lyse a larger population of bacteria. These kinetic profiles are advantageous for clinical applications targeting infections caused by the specific K3 capsule type strain, as swift phage amplification can effectively inhibit bacterial regeneration *in vivo*. Notably, strains of the K3 capsule type are among the most prevalent in clinical isolations (25). Furthermore, P11B demonstrated robust stability under maintaining high titer for at 4–42°C and across a pH range of 3–11. This stability profile compares favorably to other phages, which showed significant titer loss under similar conditions, suggesting P11B may be more suitable for formulation and storage. Therefore, the high efficiency and stability of phage P11B in lysis demonstrate significant potential and prospects for application in the treatment of clinical *A. baumannii* infections.

### Receptor-mediated specificity and kinetics of phage resistance

Our receptor recognition experiments identified three functionally related genes (*pgi*, *algC*, and *galU*) as critical determinants of resistance to phage P11B. Disruption of these genes completely abolished susceptibility to the phage in the host strain. Studies have shown that the *pgi*, *algC*, and *galU* genes are essential for all structural components and play a significant role in the synthesis of surface polysaccharides (47, 48). Mechanistic analysis revealed distinct roles for these loci: *pgi* encodes phosphoglucose isomerase, a key enzyme in the glycolysis and gluconeogenesis pathways (44); *algC* is involved in alginate biosynthesis (41), potentially modifying bacterial surface architecture and interfering with primary phage adsorption; and *galU* regulates the UDP-glucose metabolism, a precursor for cell wall polysaccharide synthesis (43), and its deficiency likely alters receptor availability. Collectively, these findings suggest that disruptions in either surface structure biogenesis or metabolic homeostasis can synergistically compromise phage-host interactions. The restoration of phage susceptibility through genetic complementation provides strong support for this model (Fig. 5). Notably, complementation of *pgi* resulted in plaques that were less clear compared to those from *algC/galU*-complemented strains. This phenotypic divergence aligns with functional categorization: while *algC* and *galU* directly mediate surface polysaccharide synthesis, which is a rate-limiting step for phage adsorption (49), *pgi* primarily modulates central carbon metabolism. These observations resonate with emerging paradigms in phage-bacteria coevolution, where surface polysaccharide diversity serves as the primary determinant of phage tropism in this work.

Phage P11B exhibits a narrow host range. It is capable of lysing all clinically isolated carbapenem-resistant *A. baumannii* (CRAB) strains belonging to the KL3 capsule type; however, strains of other capsule types demonstrate significant insensitivity to it. While this restricted tropism limits a broad-spectrum activity, it conversely enhances therapeutic safety, presenting a key advantage over broad-host phages, which indiscriminately target various *Acinetobacter* species and pose a risk of ecological disturbance. This indicates that P11B has a tailored utility in addressing regional CRAB infections. Furthermore, using the *Galleria mellonella* infection mode, the virulence tests revealed that the phage-resistant strains *pgi*-mutant, *algC*-mutant, and *galU*-mutant exhibited significantly reduced bacterial virulence compared to the wild type. In particular, the *galU*-mutant strain exhibited the most significant reduction in virulence, which is associated with the *galU* gene that encodes the virulence-related capsular polysaccharide on the bacterial surface. This has been reported in *Acinetobacter* (19) and other pathogenic bacteria (42). This phenomenon of virulence attenuation aligns with the evolutionary trade-off theory, wherein mutations conferring phage resistance reduce pathogenicity, thereby creating a 'therapeutic window' that can alleviate clinical symptoms, even if complete bacterial eradication is not achieved.

To address the issue of the narrow host spectrum of phages, the use of phage cocktails has become a widely adopted strategy (50). Phage cocktails can target various genotypes of the same bacterial species or attack multiple different bacterial species (51). Consequently, these cocktails can be designed to encompass a broader host range, thereby enhancing their efficacy in eliminating bacterial infections, particularly when treating polymorphic or polymicrobial infections that remain uncharacterized. To ensure that phages target different bacterial receptors and to avoid the emergence of phage-resistant bacteria, the rational design of cocktail therapies utilizing various phage combinations can effectively mitigate the evolution of phage resistance in target bacteria, thereby enhancing the likelihood of successful clinical treatment (52–54). P11B is a phage we isolated that specifically lyses the KL3 capsule type of *A. baumannii*. This phage enriches our library, providing valuable resources for subsequent clinical applications. The phages within our library can effectively target the vast majority of clinically isolated *A. baumannii* strains. Additionally, we are investigating cocktail therapy utilizing multiple phage combinations for the treatment of clinical infections caused by CRAB.

The combined use of phages and antibiotics represents a novel approach to combat drug-resistant bacteria. Given their fundamentally distinct antibacterial mechanisms, combination therapy has demonstrated numerous advantages and has garnered significant attention in recent years. Numerous studies have demonstrated the advantages of utilizing phages in conjunction with antibiotics. This combination can enhance the proliferation of phages, thereby increasing their sterilization efficacy (55, 56). Furthermore, for bacteria, the simultaneous acquisition of phage resistanceand antibiotic resistance is typically challenging, as evidenced by certain bacterial strains that harbor mobile genetic elements containing multiple drug resistance genes (57, 58). First, the diversity of resistance mechanisms may significantly contribute to this outcome. Bacterial resistance to phages can arise from alterations in surface receptors, metabolic changes, or adenylate methylation (59). These modifications may not directly impact the antibiotic targets, allowing phage-resistant bacteria to maintain their resistance in the presence of antibiotics. Additionally, bacteria may coexist with multiple resistance traits in environments subjected to natural selection, potentially mediated by plasmids or transposons harboring resistance genes (60). Second, the mechanisms of action of phages and antibiotics are fundamentally distinct. Consequently, the presence of phages may not necessarily alter the specific physiological state of bacteria, nor restore their sensitivity to antibiotics (61). Moreover, some lysogenic phages carry resistance genes and can facilitate the dissemination of these genes among strains (62). Lastly, the complexity of combined therapy should not be overlooked. Different bacterial species and strains exhibit varied responses to the concurrent use of phages and antibiotics, and the ecological environment and growth conditions of bacteria may influence their effectiveness. Therefore, future research should further investigate the optimal ratios of different phage-antibiotic combinations and their applicable contexts.

In summary, this study expands the resource library for phage therapy and offers novel therapeutic candidates for clinical applications. Importantly, we identified a critical trade-off between phage resistance and bacterial virulence, providing valuable insights for mitigating resistance risks in future phage-based treatments.

## Data Availability

The genetic sequences acquired during this study have been deposited into the National Center for Biotechnology Information database as a BioProject under accession number PRJNA1296866. The project contains data for *pgi*-mutant (accession number SAMN50205104), *algC*-mutant (accession number SAMN50205105), and *galU*-mutant (accession number SAMN50205106). Phage genomes were independently submitted on GenBank (P11B accession number PV851422). Source data are provided with this paper.

## SUPPLEMENTAL MATERIAL

Supplemental material spectrum.01098-25-s0001.pdf Tables S1 and S2 and primers.

ASM does not own the copyrights to Supplemental Material that may be linked to, or accessed through, an article. The authors have granted ASM a non-exclusive, world-wide license to publish the Supplemental Material files. Please contact the corresponding author directly for reuse.
