# Supplementary material for: Isolation and characterization of a novel K3-type capsule-targeting phage for the treatment of carbapenem-resistant Acinetobacter baumannii
Source: Microbiol Spectr. 2025 Nov 5;13(12):e01098-25. doi: 10.1128/spectrum.01098-25 (PMC12671153; doi:10.1128/spectrum.01098-25)
Supplement: Supplemental material — Tables S1 and S2 and primers. [file spectrum.01098-25-s0001.pdf]

Table S1 Strain information and spot test results

Strains	MLST	K Locus	OC Locus	Specimen	Isolated date	Isolated from	Biosample	SRA	Spot
TAB11B	2	KL3	OCL1	Conduct	2022.08.05	Hospital T	SAMN41855996	SRR29429968	++
GAB01B	2	KL3	OCL1	Sputum	Unknown	Hospital G	SAMN41855997	SRR29429967	++
ZAB15B	2	KL3	OCL1	Unknown	Unknown	Institution Z	SAMN41855954	SRR29430014	++
ZAB24B	2	KL3	OCL1	Unknown	Unknown	Institution Z	SAMN41855959	SRR29430009	++
ZAB37B	2	KL3	OCL1	Unknown	Unknown	Institution Z	SAMN41855970	SRR29429997	++
ZAB47B	2	KL3	OCL1	Unknown	Unknown	Institution Z	SAMN41855978	SRR29429988	++
ZAB55B	2	KL3	OCL1	Unknown	Unknown	Institution Z	SAMN41855985	SRR29429980	++
SAB01B	2	KL3	OCL1	Sputum	2020.2.13	Hospital S	SAMN41862006	SRR29430132	++
SAB03B	2	KL3	OCL1	Sputum	2020.3.25	Hospital S	SAMN41862007	SRR29430121	++
SAB04B	2	KL3	OCL1	Urine	2020.3.30	Hospital S	SAMN41862008	SRR29430110	++
ATCC17978	437	KL3	OCL2	N/A	N/A	N/A	N/A	N/A	+
FAB01B	52	KL3	OCL1	Sputum	Unknown	Institution F	SAMN50490058	SRR34900924	+
FAB02B	52	KL3	OCL1	Sputum	Unknown	Institution F	SAMN50490059	SRR34900923	+
ZAB50B	924	KL93	OCL2	Unknown	Unknown	Institution Z	SAMN41855980	SRR29429986	-
ZAB34B	2	KL9	OCL1	Unknown	Unknown	Institution Z	SAMN41855967	SRR29430000	-
ZAB02B		KL84	OCL6	Unknown	Unknown	Institution Z	SAMN41855944	SRR29430016	-
ZAB54B		KL81	OCL7	Unknown	Unknown	Institution Z	SAMN41855984	SRR29429981	-
RAB03B	2	KL77	OCL1	Environment	2021.09.03	Hospital R	SAMN41856007	SRR29429957	-
RAB04B	2	KL77	OCL1	Environment	2021.09.10	Hospital R	SAMN41856008	SRR29429956	-
RAB05B	2	KL77	OCL1	Environment	2021.09.10	Hospital R	SAMN41856009	SRR29429955	-
ZAB08B	2	KL77	OCL1	Unknown	Unknown	Institution Z	SAMN41855947	SRR29429993	-
ZAB09B	2	KL77	OCL1	Unknown	Unknown	Institution Z	SAMN41855948	SRR29429982	-
ZAB19B	2	KL77	OCL1	Unknown	Unknown	Institution Z	SAMN41855957	SRR29430011	-
ZAB26B	2	KL77	OCL1	Unknown	Unknown	Institution Z	SAMN41855960	SRR29430008	-
ZAB27B	2	KL77	OCL1	Unknown	Unknown	Institution Z	SAMN41855961	SRR29430007	-
ZAB29B	2	KL77	OCL1	Unknown	Unknown	Institution Z	SAMN41855963	SRR29430005	-
ZAB30B	2	KL77	OCL1	Unknown	Unknown	Institution Z	SAMN41855964	SRR29430003	-
ZAB35B	2	KL77	OCL1	Unknown	Unknown	Institution Z	SAMN41855968	SRR29429999	-
ZAB49B	2	KL77	OCL1	Unknown	Unknown	Institution Z	SAMN41855979	SRR29429987	-
TAB07B	2	KL72	OCL1	BALF	Unknown	Hospital T	SAMN41855993	SRR29429972	-
NAB21B	2	KL7	OCL1	Sputum	2024.01.16	Hospital N	SAMN41856003	SRR29429962	-
NAB22B	2	KL7	OCL1	Sputum	2024.01.15	Hospital N	SAMN41856004	SRR29429961	-
RAB01B	2	KL7	OCL1	Environment	2021.05.17	Hospital R	SAMN41856005	SRR29429959	-
RAB02B	2	KL7	OCL1	Environment	2021.05.28	Hospital R	SAMN41856006	SRR29429958	-
AB53777	2	KL7	OCL1	Sputum	Unknown	Hospital R	SAMN41856010	SRR29429954	-
ZAB45B	2	KL7	OCL1	Unknown	Unknown	Institution Z	SAMN41855976	SRR29429990	-
SAB18B	2	KL7	OCL1	Sputum	2022.07.18	Hospital S	SAMN41862018	SRR29430128	-
SAB20B	2	KL7	OCL1	Sputum	2022.10.10	Hospital S	SAMN41862020	SRR29430126	-
SAB21B	2	KL7	OCL1	Sputum	2022.10.15	Hospital S	SAMN41862021	SRR29430125	-

SAB22B	2	KL7	OCL1	Sputum	2022.11.06	Hospital S	SAMN41862022	SRR29430124	-
SAB23B	2	KL7	OCL1	BALF	2022.11.13	Hospital S	SAMN41862023	SRR29430123	-
SAB25B	2	KL7	OCL1	Sputum	2022.11.28	Hospital S	SAMN41862024	SRR29430122	-
SAB26B	2	KL7	OCL1	Sputum	2022.12.12	Hospital S	SAMN41862025	SRR29430120	-
SAB30B	2	KL7	OCL1	BALF	2023.02.8	Hospital S	SAMN41862028	SRR29430117	-
SAB32B	2	KL7	OCL1	BALF	2023.02.8	Hospital S	SAMN41862030	SRR29430115	-
SAB33B	2	KL7	OCL1	BALF	2023.02.8	Hospital S	SAMN41862031	SRR29430114	-
SAB34B	2	KL7	OCL1	BALF	2023.02.8	Hospital S	SAMN41862032	SRR29430113	-
SAB35B	2	KL7	OCL1	BALF	2023.02.13	Hospital S	SAMN41862033	SRR29430112	-
SAB36B	2	KL7	OCL1	BALF	2023.02.13	Hospital S	SAMN41862034	SRR29430111	-
SAB37B	2	KL7	OCL1	Sputum	2023.02.13	Hospital S	SAMN41862035	SRR29430109	-
SAB38B	2	KL7	OCL1	BALF	2023.02.13	Hospital S	SAMN41862036	SRR29430108	-
SAB39B	2	KL7	OCL1	Sputum	2023.02.13	Hospital S	SAMN41862037	SRR29430107	-
SAB40B	2	KL7	OCL1	Sputum	2023.02.13	Hospital S	SAMN41862038	SRR29430106	-
SAB41B	2	KL7	OCL1	BALF	2023.02.13	Hospital S	SAMN41862039	SRR29430105	-
SAB42B	2	KL7	OCL1	BALF	2023.02.13	Hospital S	SAMN41862040	SRR29430104	-
SAB43B	2	KL7	OCL1	BALF	2023.02.18	Hospital S	SAMN41862041	SRR29430103	-
SAB45B	2	KL7	OCL1	BALF	2023.02.20	Hospital S	SAMN41862042	SRR29430102	-
SAB46B	2	KL7	OCL1	Sputum	2023.03.04	Hospital S	SAMN41862043	SRR29430101	-
SAB48B	2	KL7	OCL1	Sputum	2023.08.07	Hospital S	SAMN41862045	SRR29430098	-
SAB49B	2	KL7	OCL1	Sputum	2023.08.08	Hospital S	SAMN41862046	SRR29430097	-
SAB50B	2	KL7	OCL1	Sputum	2023.08.08	Hospital S	SAMN41862047	SRR29430096	-
SAB53B	2	KL7	OCL1	Urine	2023.09.13	Hospital S	SAMN41862050	SRR29430093	-
SAB59B	2	KL7	OCL1	Sputum	2023.10.24	Hospital S	SAMN41862056	SRR29430086	-
ZAB46B	1463	KL69	OCL7	Unknown	Unknown	Institution Z	SAMN41855977	SRR29429989	-
ZAB03B	309	KL58	OCL1	Unknown	Unknown	Institution Z	SAMN41855945	SRR29430015	-
ZAB32B	372	KL52	OCL6	Unknown	Unknown	Institution Z	SAMN41855966	SRR29430001	-
SAB12B	164	KL47	OCL5	Sputum	2021.07.20	Hospital S	SAMN41862014	SRR29430075	-
ZAB36B	164	KL47	OCL5	Unknown	Unknown	Institution Z	SAMN41855969	SRR29429998	-
ZAB51B	282	KL47	OCL6	Unknown	Unknown	Institution Z	SAMN41855981	SRR29429985	-
ZAB56B	164	KL47	OCL5	Unknown	Unknown	Institution Z	SAMN41855986	SRR29429979	-
SAB51B	697	KL45	OCL1	BALF	2023.09.13	Hospital S	SAMN41862048	SRR29430095	-
SAB57B	697	KL45	OCL1	Sputum	2023.09.26	Hospital S	SAMN41862054	SRR29430089	-
SAB60B	697	KL45	OCL1	BALF	2023.11.17	Hospital S	SAMN41862057	SRR29430085	-
SAB65B	697	KL45	OCL1	BALF	2023.12.05	Hospital S	SAMN41862061	SRR29430081	-
ZAB16B	221	KL37	OCL7	Unknown	Unknown	Institution Z	SAMN41855955	SRR29430013	-
ZAB11B	46	KL28	OCL1	Unknown	Unknown	Institution Z	SAMN41855950	SRR29429960	-
SAB17B	1131	KL26	OCL9	Urine	2022.03.19	Hospital S	SAMN41862017	SRR29430129	-
ZAB18B	773	KL24	OCL7	Unknown	Unknown	Institution Z	SAMN41855956	SRR29430012	-
ZAB42B	1340	KL229	OCL1	Unknown	Unknown	Institution Z	SAMN41855973	SRR29429994	-
ZAB14B	93	KL220	OCL6	Unknown	Unknown	Institution Z	SAMN41855953	SRR29429951	-
NAB01B	2	KL2	OCL1	Sputum	2022.08.30	Hospital N	SAMN41724871	SRR29334559	-
NAB02B	2	KL2	OCL1	Sputum	2022.09.09	Hospital N	SAMN41726357	SRR29334558	-
NAB03B	2	KL2	OCL1	Sputum	2022.09.14	Hospital N	SAMN41726358	SRR29334548	-

NAB04B	2	KL2	OCL1	Sputum	2022.09.15	Hospital N	SAMN41726359	SRR29334547	-
NAB05B	2	KL2	OCL1	Sputum	2022.09.25	Hospital N	SAMN41726360	SRR29334546	-
NAB06B	2	KL2	OCL1	Sputum	2022.09.30	Hospital N	SAMN41726361	SRR29334545	-
NAB07B	2	KL2	OCL1	Sputum	2022.10.07	Hospital N	SAMN41726407	SRR29334544	-
NAB08B	2	KL2	OCL1	Sputum	2023.02.07	Hospital N	SAMN41753606	SRR29354114	-
NAB09B	2	KL2	OCL1	Sputum	2023.02.07	Hospital N	SAMN41753607	SRR29354115	-
NAB10B	2	KL2	OCL1	Sputum	2023.02.20	Hospital N	SAMN41855999	SRR29429965	-
NAB11B	2	KL2	OCL1	Sputum	2023.03.01	Hospital N	SAMN41753608	SRR29354113	-
NAB13B	2	KL2	OCL1	Sputum	2023.02.22	Hospital N	SAMN41856000	SRR29429964	-
NAB14B	2	KL2	OCL1	Sputum	2023.03.10	Hospital N	SAMN41856001	SRR33649602	-
NAB17B	2	KL2	OCL1	Sputum	2023.06.23	Hospital N	SAMN41726563	SRR33649601	-
NAB18B		KL2	OCL1	Sputum	2023.09.17	Hospital N	SAMN41856002	SRR29429963	-
GAB02B	2	KL2	OCL1	Sputum	Unknown	Hospital G	SAMN41855998	SRR29429966	-
TAB01B	2	KL2	OCL1	Sputum	2021.12.23	Hospital T	SAMN41855987	SRR29429978	-
TAB04B	2	KL2	OCL1	BALF	2021.12.18	Hospital T	SAMN41855990	SRR29429975	-
TAB05B	2	KL2	OCL1	Ascites	2022.01.14	Hospital T	SAMN41855991	SRR29429974	-
TAB06B	2	KL2	OCL1	BALF	2022.01.16	Hospital T	SAMN41855992	SRR29429973	-
TAB09B	2	KL2	OCL1	Sputum	2022.05.18	Hospital T	SAMN41726601	SRR29334550	-
TAB10B	2	KL2	OCL1	Sputum	2022.06.26	Hospital T	SAMN41855995	SRR29429969	-
ZAB01B	2	KL2	OCL1	Unknown	Unknown	Institution Z	SAMN41726603	SRR29334549	-
ZAB12B	2	KL2	OCL1	Unknown	Unknown	Institution Z	SAMN41855951	SRR29429953	-
ZAB40B	2	KL2	OCL1	Unknown	Unknown	Institution Z	SAMN41855972	SRR29429995	-
ZAB53B	2	KL2	OCL1	Unknown	Unknown	Institution Z	SAMN41855983	SRR29429983	-
SAB05B	2	KL2	OCL1	Sputum	2020.3.30	Hospital S	SAMN41862009	SRR29430099	-
SAB09B	2	KL2	OCL1	Secreta	2021.1.7	Hospital S	SAMN41862011	SRR29430078	-
SAB10B	2	KL2	OCL1	Sputum	2021.4.16	Hospital S	SAMN41862012	SRR29430077	-
SAB11B	2	KL2	OCL1	Sputum	2021.4.16	Hospital S	SAMN41862013	SRR29430076	-
SAB14B	2	KL2	OCL1	BALF	2021.10.11	Hospital S	SAMN41862015	SRR29430131	-
SAB15B	2	KL2	OCL1	Urine	2021.10.13	Hospital S	SAMN41862016	SRR29430130	-
SAB19B	2	KL2	OCL1	Sputum	2022.09.10	Hospital S	SAMN41862019	SRR29430127	-
SAB27B	2	KL2	OCL1	Sputum	2022.12.12	Hospital S	SAMN41862026	SRR29430119	-
SAB29B	2	KL2	OCL1	BALF	2023.02.8	Hospital S	SAMN41862027	SRR29430118	-
SAB31B	2	KL2	OCL1	Blood	2023.02.8	Hospital S	SAMN41862029	SRR29430116	-
SAB47B	2	KL2	OCL1	Sputum	2023.05.25	Hospital S	SAMN41862044	SRR29430100	-
SAB52B	2	KL2	OCL1	BALF	2023.09.13	Hospital S	SAMN41862049	SRR29430094	-
SAB54B	2	KL2	OCL1	BALF	2023.09.22	Hospital S	SAMN41862051	SRR29430092	-
SAB55B	2	KL2	OCL1	BALF	2023.09.22	Hospital S	SAMN41862052	SRR29430091	-
SAB56B	697	KL2	OCL1	BALF	2023.09.26	Hospital S	SAMN41862053	SRR29430090	-
SAB58B	2	KL2	OCL1	Sputum	2023.10.17	Hospital S	SAMN41862055	SRR29430087	-
SAB61B	2	KL2	OCL1	BALF	2023.11.17	Hospital S	SAMN41862058	SRR29430084	-
SAB62B	2	KL2	OCL1	Sputum	2023.11.17	Hospital S	SAMN41862059	SRR29430083	-
SAB63B	2	KL2	OCL1	Sputum	2023.11.17	Hospital S	SAMN41862060	SRR29430082	-
SAB66B	2	KL2	OCL1	Sputum	2023.12.11	Hospital S	SAMN41862062	SRR29430080	-
SAB67B	2	KL2	OCL1	Ascites	2023.12.11	Hospital S	SAMN41862063	SRR29430079	-

SAB57B	697	KL45	OCL1	Sputum	2023.09.26	Hospital S	SAMN41862054	SRR29430089	-
SAB59B	2	KL7	OCL1	Sputum	2023.10.24	Hospital S	SAMN41862056	SRR29430086	-
SAB60B	697	KL45	OCL1	BALF	2023.11.17	Hospital S	SAMN41862057	SRR29430085	-
ZAB13B	1264	KL197	OCL1	Unknown	Unknown	Institution Z	SAMN41855952	SRR29429952	-
ZAB31B	1405	KL176	OCL5	Unknown	Unknown	Institution Z	SAMN41855965	SRR29430002	-
RAB06B	2	KL160	OCL1	Sputum	Unknown	Hospital R	SAMN41862005	SRR29430133	-
TAB08B	2	KL160	OCL1	Sputum	2022.05.02	Hospital T	SAMN41855994	SRR29429970	-
ZAB10B	2	KL160	OCL1	Unknown	Unknown	Institution Z	SAMN41855949	SRR29429971	-
ZAB21B	33	KL14	OCL6	Unknown	Unknown	Institution Z	SAMN41855958	SRR29430010	-
SAB07B		KL132	OCL7	Sputum	2020.5.28	Hospital S	SAMN41862010	SRR29430088	-
ZAB38B	213	KL128	OCL6	Unknown	Unknown	Institution Z	SAMN41855971	SRR29429996	-
ZAB43B	445	KL128	OCL7	Unknown	Unknown	Institution Z	SAMN41855974	SRR29429992	-
TAB02B	2	KL125	OCL1	BALF	2021.12.31	Hospital T	SAMN41855988	SRR29429977	-
ZAB28B	2	KL125	OCL1	Unknown	Unknown	Institution Z	SAMN41855962	SRR29430006	-
ZAB44B	336	KL125	OCL5	Unknown	Unknown	Institution Z	SAMN41855975	SRR29429991	-
ZAB52B	2	KL125	OCL1	Unknown	Unknown	Institution Z	SAMN41855982	SRR29429984	-
ZAB04B	93	KL111	OCL6	Unknown	Unknown	Institution Z	SAMN41855946	SRR29430004	-

Note: Hospital S: Shenzhen University General Hospital; Hospital N: Southern University of Science and Technology Hospital; Hospital T: Shenzhen Third People's Hospital; Hospital G: The First Affiliated Hospital of Guangzhou Medical University; Hospital R: Shenzhen People's Hospital; Institution F: Fudan University; Institution Z: Sun Yat-Sen University.

++: Presence of clear and translucent plaque; +: Presence of plaque but unclear; -: Absence of plaque.

primers of target genes (*pgi, algC, galU*)

pgi F: ggagaaaggatcttacatgagtaaatctatcgagaagtttcctaaagag

pgi R: taccgagggatttattagccatcgacttttcctaataaaattttaatcaaacc

algC F: ggagaaaggatcttacatgactactttaacgtgttttaaagcttatgatatcc

algC R: tggtaccgagggatttattagttctgaattaatccagtcaactcatttac

galU F: ggagaaaggatcttacatgattaaaaaagcagttttaccagtagccg

galU R: ggtaccgagggatttattataatttaagttcctgaatcaactgtttaaaatcatttcc

Table S2 Compared with TAB11B, the K locus mutations of other K3 strains

Strain	POS	TYPE	REF	ALT	EVIDENCE	NT_POS	AA_POS	EFFECT	GENE	PRODUCT
ATCC17978	1894	complex	AACAGAA	GACCGTG	GACCGTG:16 AACAGAA:0	294/2187	96/728	missense	ptk	Tyrosine-protein kinase ptk
ATCC17978	3512	snp	T	A	A:20 T:0	229/1101	77/366	missense		hypothetical protein
ATCC17978	4187	complex	AGTAA	GGTAG	GGTAG:20 AGTAA:0	87/1296	29/431	missense	wbpA	UDP-N-acetyl-D-glucosamine 6-dehydrogenase
ATCC17978	7290	complex	GT	AG	AG:20 GT:0	337/1080	113/359	missense	wbpE	UDP-2-acetamido-2-deoxy-3-oxo-D-glucuronate aminotransferase
ATCC17978	7375	snp	C	T	T:20 C:0	422/1080	141/359	missense	wbpE	UDP-2-acetamido-2-deoxy-3-oxo-D-glucuronate aminotransferase
ATCC17978	7452	snp	G	A	A:20 G:0	499/1080	167/359	missense	wbpE	UDP-2-acetamido-2-deoxy-3-oxo-D-glucuronate aminotransferase
ATCC17978	7743	snp	G	A	A:20 G:0	790/1080	264/359	missense	wbpE	UDP-2-acetamido-2-deoxy-3-oxo-D-glucuronate aminotransferase
ATCC17978	11555	snp	T	A	A:20 T:0	141/1092	47/363	missense		hypothetical protein
ATCC17978	11994	snp	A	G	G:20 A:0	580/1092	194/363	missense		hypothetical protein
ATCC17978	12393	snp	T	C	C:20 T:0	979/1092	327/363	missense		hypothetical protein
ATCC17978	13054	snp	G	A	A:20 G:0	466/1041	156/346	missense		hypothetical protein
ATCC17978	13780	snp	T	C	C:20 T:0	148/1035	50/344	missense		glycosyltransferase
ATCC17978	14132	snp	G	A	A:20 G:0	500/1035	167/344	missense		glycosyltransferase
ATCC17978	14140	complex	AA	GG	GG:20 AA:0	508/1035	170/344	missense		glycosyltransferase
ATCC17978	14277	snp	T	G	G:20 T:0	645/1035	215/344	missense		glycosyltransferase
ATCC17978	14832	snp	G	C	C:20 G:0	159/828	53/275	missense	wbbD	UDP-Gal:alpha-D-GlcNAc-diphosphoundecaprenol beta-1,3-galactosyltransferase
ATCC17978	14851	snp	G	A	A:20 G:0	178/828	60/275	missense	wbbD	UDP-Gal:alpha-D-GlcNAc-diphosphoundecaprenol beta-1,3-galactosyltransferase
ATCC17978	15104	snp	T	A	A:20 T:0	431/828	144/275	missense	wbbD	UDP-Gal:alpha-D-GlcNAc-diphosphoundecaprenol beta-1,3-galactosyltransferase
ATCC17978	16113	complex	CGTA	TATT	TATT:18 CGTA:0	600/621	200/206	missense	epsL	putative sugar transferase EpsL
ATCC17978	16577	snp	C	A	A:20 C:0	418/876	140/291	missense	galU	UTP--glucose-1-phosphate uridylyltransferase
ATCC17978	16601	snp	T	G	G:20 T:0	442/876	148/291	missense	galU	UTP--glucose-1-phosphate uridylyltransferase
ATCC17978	16885	snp	C	A	A:20 C:0	726/876	242/291	missense	galU	UTP--glucose-1-phosphate uridylyltransferase
ATCC17978	16893	snp	T	C	C:20 T:0	734/876	245/291	missense	galU	UTP--glucose-1-phosphate uridylyltransferase
ATCC17978	17502	snp	G	A	A:20 G:0	352/1263	118/420	missense		UDP-glucose dehydrogenase
ATCC17978	18210	snp	A	G	G:20 A:0	1060/1263	354/420	missense		UDP-glucose dehydrogenase

ATCC17978	18315	snp	T	G	G:20 T:0	1165/1263	389/420	missense		UDP-glucose dehydrogenase
ATCC17978	19347	snp	A	G	G:20 A:0	938/1671	313/556	missense	pgi	Glucose-6-phosphate isomerase
ATCC17978	20021	snp	A	G	G:20 A:0	1612/1671	538/556	missense	pgi	Glucose-6-phosphate isomerase
ATCC17978	21162	snp	T	C	C:20 T:0	1342/1371	448/456	missense	algC	Phosphomannomutase/phosphoglucomutase
ATCC17978	21315	complex	TC	CG	CG:20 TC:0	1189/1371	396/456	missense	algC	Phosphomannomutase/phosphoglucomutase
ATCC17978	21435	snp	T	C	C:20 T:0	1069/1371	357/456	missense	algC	Phosphomannomutase/phosphoglucomutase
FAB01B	1492	complex	GGCCACAGC	TGCTACTGA	TGCTACTGA:11 GGCCACAGC:0	696/2187	230/728	missense	ptk	Tyrosine-protein kinase ptk
FAB01B	1507	complex	CGCA	TGTG	TGTG:11 CGCA:0	681/2187	226/728	missense	ptk	Tyrosine-protein kinase ptk
FAB01B	1515	complex	TA	GT	GT:11 TA:0	673/2187	224/728	missense	ptk	Tyrosine-protein kinase ptk
FAB01B	1529	complex	ATA	GCT	GCT:11 ATA:0	659/2187	219/728	missense	ptk	Tyrosine-protein kinase ptk
FAB01B	1536	complex	AAGATA	TTGATT	TTGATT:11 AAGATA:0	652/2187	216/728	missense	ptk	Tyrosine-protein kinase ptk
FAB01B	1772	snp	C	T	T:10 C:0	416/2187	139/728	missense	ptk	Tyrosine-protein kinase ptk
FAB01B	1783	complex	AATG	TAAA	TAAA:10 AATG:0	405/2187	134/728	missense	ptk	Tyrosine-protein kinase ptk
FAB01B	1796	snp	G	T	T:11 G:0	392/2187	131/728	missense	ptk	Tyrosine-protein kinase ptk
FAB01B	1821	snp	C	T	T:13 C:0	367/2187	123/728	missense	ptk	Tyrosine-protein kinase ptk
FAB01B	1894	complex	AACAGAA	GACCGTG	GACCGTG:11 AACAGAA:0	294/2187	96/728	missense	ptk	Tyrosine-protein kinase ptk
FAB01B	2567	snp	A	T	T:20 A:0	69/429	23/142	missense	ptp	Low molecular weight protein-tyrosine-phosphatase Ptp
FAB01B	2820	complex	GGTTGTGCGG	AGTAACATGA	AGTAACATGA:16 GGTTGTGCGG:0	921/1101	304/366	missense		hypothetical protein
FAB01B	2913	complex	GCTTAG	AGTTAA	AGTTAA:17 GCTTAG:0	828/1101	275/366	missense		hypothetical protein
FAB01B	3485	complex	CATCACCTGGA	TATCGCCAGGG	TATCGCCAGGG:11 CATCACCTGGA:0	256/1101	82/366	missense		hypothetical protein
FAB01B	3512	complex	TTTGTTGAT	ACTGCTGCG	ACTGCTGCG:11 TTTGTTGAT:0	229/1101	74/366	missense		hypothetical protein
FAB01B	5871	snp	G	C	C:20 G:0	445/951	149/316	missense	wbpB	UDP-N-acetyl-2-amino-2-deoxy-D-glucuronate oxidase
FAB01B	6430	snp	A	C	C:20 A:0	57/579	19/192	missense	wbpD	UDP-2-acetamido-3-amino-2, 3-dideoxy-D-glucuronate N-acetyltransferase
FAB01B	6440	snp	G	A	A:20 G:0	67/579	23/192	missense	wbpD	UDP-2-acetamido-3-amino-2, 3-dideoxy-D-glucuronate N-acetyltransferase
FAB01B	6731	snp	G	A	A:20 G:0	358/579	120/192	missense	wbpD	UDP-2-acetamido-3-amino-2, 3-dideoxy-D-glucuronate N-acetyltransferase
FAB01B	6755	snp	A	G	G:20 A:0	382/579	128/192	missense	wbpD	UDP-2-acetamido-3-amino-2, 3-dideoxy-D-glucuronate N-acetyltransferase
FAB01B	7008	snp	G	A	A:20 G:0	55/1080	19/359	missense	wbpE	UDP-2-acetamido-2-deoxy-3-oxo-D-glucuronate aminotransferase
FAB01B	7177	snp	A	G	G:20 A:0	224/1080	75/359	missense	wbpE	UDP-2-acetamido-2-deoxy-3-oxo-D-glucuronate aminotransferase

FAB01B	7290	snp	G	A	A:20 G:0	337/1080	113/359	missense	wbpE	UDP-2-acetamido-2-deoxy-3-oxo-D-glucuronate aminotransferase
FAB01B	7743	snp	G	A	A:20 G:0	790/1080	264/359	missense	wbpE	UDP-2-acetamido-2-deoxy-3-oxo-D-glucuronate aminotransferase
FAB01B	7748	snp	G	T	T:20 G:0	795/1080	265/359	missense	wbpE	UDP-2-acetamido-2-deoxy-3-oxo-D-glucuronate aminotransferase
FAB01B	7779	complex	GCGC	TCGT	TCGT:19 GCGC:0	826/1080	276/359	missense	wbpE	UDP-2-acetamido-2-deoxy-3-oxo-D-glucuronate aminotransferase
FAB01B	7993	snp	C	T	T:20 C:0	1040/1080	347/359	missense	wbpE	UDP-2-acetamido-2-deoxy-3-oxo-D-glucuronate aminotransferase
FAB01B	8233	snp	C	G	G:20 C:0	166/1353	56/450	missense		hypothetical protein
FAB01B	12393	snp	T	C	C:20 T:0	979/1092	327/363	missense		hypothetical protein
FAB01B	12422	snp	T	A	A:20 T:0	1008/1092	336/363	missense		hypothetical protein
FAB01B	13054	snp	G	A	A:20 G:0	466/1041	156/346	missense		hypothetical protein
FAB01B	13780	snp	T	C	C:20 T:0	148/1035	50/344	missense		glycosyltransferase
FAB01B	14132	snp	G	A	A:20 G:0	500/1035	167/344	missense		glycosyltransferase
FAB01B	14239	snp	C	A	A:20 C:0	607/1035	203/344	missense		glycosyltransferase
FAB01B	14268	snp	A	T	T:20 A:0	636/1035	212/344	missense		glycosyltransferase
FAB01B	14277	snp	T	G	G:20 T:0	645/1035	215/344	missense		glycosyltransferase
FAB01B	14301	snp	G	T	T:20 G:0	669/1035	223/344	missense		glycosyltransferase
FAB01B	14526	snp	T	A	A:20 T:0	894/1035	298/344	missense		glycosyltransferase
FAB01B	14542	snp	G	A	A:20 G:0	910/1035	304/344	missense		glycosyltransferase
FAB01B	14551	snp	G	C	C:20 G:0	919/1035	307/344	missense		glycosyltransferase
FAB01B	14605	snp	G	T	T:20 G:0	973/1035	325/344	missense		glycosyltransferase
FAB01B	14637	snp	A	T	T:20 A:0	1005/1035	335/344	missense		glycosyltransferase
FAB01B	14851	snp	G	A	A:20 G:0	178/828	60/275	missense	wbbD	UDP-Gal:alpha-D-GlcNAc-diphosphoundecaprenol beta-1,3-galactosyltransferase
FAB01B	15104	complex	TGGAA	AGGAG	AGGAG:18 TGGAA:0	431/828	144/275	missense	wbbD	UDP-Gal:alpha-D-GlcNAc-diphosphoundecaprenol beta-1,3-galactosyltransferase
FAB01B	16113	complex	CGTA	TATT	TATT:18 CGTA:0	600/621	200/206	missense	epsL	putative sugar transferase EpsL
FAB01B	16527	snp	A	T	T:20 A:0	368/876	123/291	missense	galU	UTP--glucose-1-phosphate uridylyltransferase
FAB01B	16577	snp	C	A	A:20 C:0	418/876	140/291	missense	galU	UTP--glucose-1-phosphate uridylyltransferase
FAB01B	16601	snp	T	G	G:20 T:0	442/876	148/291	missense	galU	UTP--glucose-1-phosphate uridylyltransferase
FAB01B	16885	snp	C	A	A:20 C:0	726/876	242/291	missense	galU	UTP--glucose-1-phosphate uridylyltransferase
FAB01B	16893	snp	T	C	C:20 T:0	734/876	245/291	missense	galU	UTP--glucose-1-phosphate uridylyltransferase
FAB01B	18210	snp	A	G	G:20 A:0	1060/1263	354/420	missense		UDP-glucose dehydrogenase
FAB01B	18315	snp	T	G	G:20 T:0	1165/1263	389/420	missense		UDP-glucose dehydrogenase

FAB01B	19180	snp	G	T	T:20 G:0	771/1671	257/556	missense	pgi	Glucose-6-phosphate isomerase
FAB01B	19347	snp	A	G	G:20 A:0	938/1671	313/556	missense	pgi	Glucose-6-phosphate isomerase
FAB01B	19697	snp	A	T	T:20 A:0	1288/1671	430/556	missense	pgi	Glucose-6-phosphate isomerase
FAB01B	20226	snp	A	G	G:18 A:0	154/1017	52/338	missense	galE	UDP-glucose 4-epimerase
FAB01B	20275	snp	A	G	G:18 A:0	203/1017	68/338	missense	galE	UDP-glucose 4-epimerase
FAB01B	21162	snp	T	C	C:19 T:0	1342/1371	448/456	missense	algC	Phosphomannomutase/phosphoglucomutase
FAB01B	21192	complex	GG	TA	TA:19 GG:0	1312/1371	437/456	missense	algC	Phosphomannomutase/phosphoglucomutase
FAB01B	21198	snp	C	T	T:19 C:0	1306/1371	436/456	missense	algC	Phosphomannomutase/phosphoglucomutase
FAB01B	21262	complex	CGCA	TGTG	TGTG:18 CGCA:0	1242/1371	413/456	missense	algC	Phosphomannomutase/phosphoglucomutase
FAB01B	21359	snp	G	A	A:18 G:0	1145/1371	382/456	missense	algC	Phosphomannomutase/phosphoglucomutase
FAB01B	21435	snp	T	C	C:18 T:0	1069/1371	357/456	missense	algC	Phosphomannomutase/phosphoglucomutase
FAB02B	1492	complex	GGCCACAGC	TGCTACTGA	TGCTACTGA:11 GGCCACAGC:0	696/2187	230/728	missense	ptk	Tyrosine-protein kinase ptk
FAB02B	1507	complex	CGCA	TGTG	TGTG:11 CGCA:0	681/2187	226/728	missense	ptk	Tyrosine-protein kinase ptk
FAB02B	1515	complex	TA	GT	GT:11 TA:0	673/2187	224/728	missense	ptk	Tyrosine-protein kinase ptk
FAB02B	1529	complex	ATA	GCT	GCT:11 ATA:0	659/2187	219/728	missense	ptk	Tyrosine-protein kinase ptk
FAB02B	1536	complex	AAGATA	TTGATT	TTGATT:11 AAGATA:0	652/2187	216/728	missense	ptk	Tyrosine-protein kinase ptk
FAB02B	1772	snp	C	T	T:10 C:0	416/2187	139/728	missense	ptk	Tyrosine-protein kinase ptk
FAB02B	1783	complex	AATG	TAAA	TAAA:10 AATG:0	405/2187	134/728	missense	ptk	Tyrosine-protein kinase ptk
FAB02B	1796	snp	G	T	T:11 G:0	392/2187	131/728	missense	ptk	Tyrosine-protein kinase ptk
FAB02B	1821	snp	C	T	T:13 C:0	367/2187	123/728	missense	ptk	Tyrosine-protein kinase ptk
FAB02B	1894	complex	AACAGAA	GACCGTG	GACCGTG:11 AACAGAA:0	294/2187	96/728	missense	ptk	Tyrosine-protein kinase ptk
FAB02B	2567	snp	A	T	T:20 A:0	69/429	23/142	missense	ptp	Low molecular weight protein-tyrosine-phosphatase Ptp
FAB02B	2820	complex	GGTTGTGCGG	AGTAACATGA	AGTAACATGA:16 GGTTGTGCGG:0	921/1101	304/366	missense		hypothetical protein
FAB02B	2913	complex	GCTTAG	AGTTAA	AGTTAA:17 GCTTAG:0	828/1101	275/366	missense		hypothetical protein
FAB02B	3485	complex	CATCACCTGGA	TATCGCCAGGG	TATCGCCAGGG:11 CATCACCTGGA:0	256/1101	82/366	missense		hypothetical protein
FAB02B	3512	complex	TTTGTTGAT	ACTGCTGCG	ACTGCTGCG:11 TTTGTTGAT:0	229/1101	74/366	missense		hypothetical protein
FAB02B	5871	snp	G	C	C:20 G:0	445/951	149/316	missense	wbpB	UDP-N-acetyl-2-amino-2-deoxy-D-glucuronate oxidase
FAB02B	6430	snp	A	C	C:20 A:0	57/579	19/192	missense	wbpD	UDP-2-acetamido-3-amino-2, 3-dideoxy-D-glucuronate N-acetyltransferase
FAB02B	6440	snp	G	A	A:20 G:0	67/579	23/192	missense	wbpD	UDP-2-acetamido-3-amino-2, 3-dideoxy-D-glucuronate N-acetyltransferase
FAB02B	6731	snp	G	A	A:20 G:0	358/579	120/192	missense	wbpD	UDP-2-acetamido-3-amino-2, 3-dideoxy-D-glucuronate N-acetyltransferase
FAB02B	6755	snp	A	G	G:20 A:0	382/579	128/192	missense	wbpD	UDP-2-acetamido-3-amino-2, 3-dideoxy-D-glucuronate N-acetyltransferase

FAB02B	7008	snp	G	A	A:20 G:0	55/1080	19/359	missense	wbpE	UDP-2-acetamido-2-deoxy-3-oxo-D-glucuronate aminotransferase
FAB02B	7177	snp	A	G	G:20 A:0	224/1080	75/359	missense	wbpE	UDP-2-acetamido-2-deoxy-3-oxo-D-glucuronate aminotransferase
FAB02B	7290	snp	G	A	A:20 G:0	337/1080	113/359	missense	wbpE	UDP-2-acetamido-2-deoxy-3-oxo-D-glucuronate aminotransferase
FAB02B	7743	snp	G	A	A:20 G:0	790/1080	264/359	missense	wbpE	UDP-2-acetamido-2-deoxy-3-oxo-D-glucuronate aminotransferase
FAB02B	7748	snp	G	T	T:20 G:0	795/1080	265/359	missense	wbpE	UDP-2-acetamido-2-deoxy-3-oxo-D-glucuronate aminotransferase
FAB02B	7779	complex	GCGC	TCGT	TCGT:19 GCGC:0	826/1080	276/359	missense	wbpE	UDP-2-acetamido-2-deoxy-3-oxo-D-glucuronate aminotransferase
FAB02B	7993	snp	C	T	T:20 C:0	1040/1080	347/359	missense	wbpE	UDP-2-acetamido-2-deoxy-3-oxo-D-glucuronate aminotransferase
FAB02B	8233	snp	C	G	G:20 C:0	166/1353	56/450	missense		hypothetical protein
FAB02B	12393	snp	T	C	C:20 T:0	979/1092	327/363	missense		hypothetical protein
FAB02B	12422	snp	T	A	A:20 T:0	1008/1092	336/363	missense		hypothetical protein
FAB02B	13054	snp	G	A	A:20 G:0	466/1041	156/346	missense		hypothetical protein
FAB02B	13780	snp	T	C	C:20 T:0	148/1035	50/344	missense		glycosyltransferase
FAB02B	14132	snp	G	A	A:20 G:0	500/1035	167/344	missense		glycosyltransferase
FAB02B	14239	snp	C	A	A:20 C:0	607/1035	203/344	missense		glycosyltransferase
FAB02B	14268	snp	A	T	T:20 A:0	636/1035	212/344	missense		glycosyltransferase
FAB02B	14277	snp	T	G	G:20 T:0	645/1035	215/344	missense		glycosyltransferase
FAB02B	14301	snp	G	T	T:20 G:0	669/1035	223/344	missense		glycosyltransferase
FAB02B	14526	snp	T	A	A:20 T:0	894/1035	298/344	missense		glycosyltransferase
FAB02B	14542	snp	G	A	A:20 G:0	910/1035	304/344	missense		glycosyltransferase
FAB02B	14551	snp	G	C	C:20 G:0	919/1035	307/344	missense		glycosyltransferase
FAB02B	14605	snp	G	T	T:20 G:0	973/1035	325/344	missense		glycosyltransferase
FAB02B	14637	snp	A	T	T:20 A:0	1005/1035	335/344	missense		glycosyltransferase
FAB02B	14851	snp	G	A	A:20 G:0	178/828	60/275	missense	wbbD	UDP-Gal:alpha-D-GlcNAc-diphosphoundecaprenol beta-1,3-galactosyltransferase
FAB02B	15104	complex	TGGAA	AGGAG	AGGAG:18 TGGAA:0	431/828	144/275	missense	wbbD	UDP-Gal:alpha-D-GlcNAc-diphosphoundecaprenol beta-1,3-galactosyltransferase
FAB02B	16113	complex	CGTA	TATT	TATT:18 CGTA:0	600/621	200/206	missense	epsL	putative sugar transferase EpsL
FAB02B	16527	snp	A	T	T:20 A:0	368/876	123/291	missense	galU	UTP--glucose-1-phosphate uridylyltransferase
FAB02B	16577	snp	C	A	A:20 C:0	418/876	140/291	missense	galU	UTP--glucose-1-phosphate uridylyltransferase
FAB02B	16601	snp	T	G	G:20 T:0	442/876	148/291	missense	galU	UTP--glucose-1-phosphate uridylyltransferase
FAB02B	16885	snp	C	A	A:20 C:0	726/876	242/291	missense	galU	UTP--glucose-1-phosphate uridylyltransferase

FAB02B	16893	snp	T	C	C:20 T:0	734/876	245/291	missense	galU	UTP--glucose-1-phosphate uridylyltransferase
FAB02B	18210	snp	A	G	G:20 A:0	1060/1263	354/420	missense		UDP-glucose dehydrogenase
FAB02B	18315	snp	T	G	G:20 T:0	1165/1263	389/420	missense		UDP-glucose dehydrogenase
FAB02B	19180	snp	G	T	T:20 G:0	771/1671	257/556	missense	pgi	Glucose-6-phosphate isomerase
FAB02B	19347	snp	A	G	G:20 A:0	938/1671	313/556	missense	pgi	Glucose-6-phosphate isomerase
FAB02B	19697	snp	A	T	T:20 A:0	1288/1671	430/556	missense	pgi	Glucose-6-phosphate isomerase
FAB02B	20226	snp	A	G	G:18 A:0	154/1017	52/338	missense	galE	UDP-glucose 4-epimerase
FAB02B	20275	snp	A	G	G:18 A:0	203/1017	68/338	missense	galE	UDP-glucose 4-epimerase
FAB02B	21162	snp	T	C	C:19 T:0	1342/1371	448/456	missense	algC	Phosphomannomutase/phosphoglucomutase
FAB02B	21192	complex	GG	TA	TA:19 GG:0	1312/1371	437/456	missense	algC	Phosphomannomutase/phosphoglucomutase
FAB02B	21198	snp	C	T	T:19 C:0	1306/1371	436/456	missense	algC	Phosphomannomutase/phosphoglucomutase
FAB02B	21262	complex	CGCA	TGTG	TGTG:18 CGCA:0	1242/1371	413/456	missense	algC	Phosphomannomutase/phosphoglucomutase
FAB02B	21359	snp	G	A	A:18 G:0	1145/1371	382/456	missense	algC	Phosphomannomutase/phosphoglucomutase
FAB02B	21435	snp	T	C	C:18 T:0	1069/1371	357/456	missense	algC	Phosphomannomutase/phosphoglucomutase
SAB01B	13372	ins	G	GT	GT:18 G:0	793/1041	265/346	frameshift		hypothetical protein
ZAB47B	486	del	TC	T	T:20 TC:0	1701/2187	567/728	frameshift	ptk	Tyrosine-protein kinase ptk
